# Mammographic density as an image-based biomarker of therapy response in neoadjuvant-treated breast cancer patients

**DOI:** 10.1007/s10552-020-01379-w

**Published:** 2020-12-30

**Authors:** Ida Skarping, Daniel Förnvik, Uffe Heide-Jørgensen, Hanna Sartor, Per Hall, Sophia Zackrisson, Signe Borgquist

**Affiliations:** 1 Division of Oncology and Pathology, Department of Clinical Sciences, Lund University, Skane University Hospital, Barngatan 4, 221 85 Lund, Sweden; 2 Department of Translational Medicine, Medical Radiation Physics, Lund University, Skane University Hospital, Malmö, Sweden; 3 Department of Clinical Epidemiology, Aarhus University Hospital, Aarhus, Denmark; 4 Department of Translational Medicine, Diagnostic Radiology, Lund University, Skane University Hospital, Lund and Malmö, Sweden; 5 Department of Medical Epidemiology and Biostatistics, Karolinska Institute, Solna, Sweden; 6 Department of Oncology, Södersjukhuset, Stockholm, Sweden; 7 Department of Oncology, Aarhus University Hospital, Aarhus, Denmark

**Keywords:** Breast cancer, Mammography, Breast density, Neoadjuvant chemotherapy

## Abstract

**Purpose:**

Personalized cancer treatment requires predictive biomarkers, including image-based biomarkers. Breast cancer (BC) patients receiving neoadjuvant chemotherapy (NACT) are in a clinically vulnerable situation with the tumor present. This study investigated whether mammographic density (MD), assessed pre-NACT, is predictive of pathological complete response (pCR).

**Methods:**

A total of 495 BC patients receiving NACT in Sweden 2005–2019 were included, merged from two different cohorts. Cohort 1 was retrospectively collected (*n* = 295) and cohort 2 was prospectively collected (*n* = 200). Mammograms were scored for MD pre-NACT according to the Breast Imaging-Reporting and Data System (BI-RADS), 5th Edition. The association between MD and accomplishing pCR post-NACT was analyzed using logistic regression models—for the whole cohort, stratified by menopausal status, and in different St. Gallen surrogate subtypes.

**Results:**

In comparison to patients with low MD (BI-RADS a), the multivariable-adjusted odds ratio (OR) of accomplishing pCR following NACT was on a descending scale: 0.62 (95% confidence interval (CI) 0.24–1.57), 0.38 (95% CI 0.14–1.02), and 0.32 (95% CI 0.09–1.08) for BI-RADS b, c, and d, respectively. For premenopausal patients selectively, the corresponding point estimates were lower, although wider CIs: 0.31 (95% CI 0.06–1.62), 0.24 (95% CI 0.04–1.27), and 0.13 (95% CI 0.02–0.88). Subgroup analyses based on BC subtypes resulted in imprecise estimates, i.e., wide CIs.

**Conclusions:**

It seemed as though patients with higher MD at baseline were less likely to reach pCR after NACT—a finding more pronounced in premenopausal women. Larger multicenter studies are needed to enable analyses and interpretation for different BC subtypes.

**Supplementary Information:**

The online version of this article (10.1007/s10552-020-01379-w) contains supplementary material, which is available to authorized users.

## Introduction

Breast cancer (BC) is the most common cancer in women, with more than two million new cases each year; it accounts for almost one in four cancer cases in women worldwide [1]. As many as 12% of the female population is affected by BC at some point during their lifetimes [2]. Mammographic density (MD) refers to the radiologically dense epithelium and stroma identified in a mammogram [3]. MD is one of the strongest risk factors for BC, after aging and genetic mutations [2]. Although it is impossible to establish the cause of BC in specific cases, it is estimated that almost 30% of premenopausal and 15% of postmenopausal BC can be attributed to high MD alone [4].

Due to the cost and complexity of gene expression [5], as is mandatory for the intrinsic system developed by Perou and Sorlie [6], a surrogate classification system was developed based on the expression of a selected set of markers [7]: estrogen receptor (ER), progesterone receptor (PR), human epidermal growth factor receptor 2 (HER2), and the proliferation marker Ki67, as included in the 2011 St. Gallen International Consensus [8], all in an attempt to provide personalized cancer treatment. Currently, treatment decisions, preferably undertaken by a multidisciplinary team, are most often based on the immunohistochemistry (IHC)-based St. Gallen surrogate subtypes, histological grade, TNM stage (partly derived from radiology), and patient characteristics and preferences [9, 10].

The indication for neoadjuvant chemotherapy (NACT) in BC has broadened [9]. Hence, a larger proportion of patients receive their systemic treatment before surgery instead of after surgery, with equal long-term survival [11]. The proportion of BC patients accomplishing complete pathological response (pCR) following NACT, considered advantageous prognostically and used as a surrogate marker for long-term survival [12], varies depending on BC surrogate subtype [12]. High rates of pCR are most often achieved for the triple negative and HER2 positive subtypes [12]. However, because the majority of patients do not accomplish pCR [12], additional treatment predictive biomarkers, e.g., imaging biomarkers, are needed to offer future BC patients more individualized treatment and select the patients most likely to respond to NACT.

Previous studies [13–15], including two studies performed by our group [15, 16], have shown inconsistent results regarding MD as a predictive biomarker of response to NACT. One study shows no association between MD and pCR [14], whereas two other studies find an association between low MD and increased likelihood of pCR [13, 15]. In this study, based on two merged cohorts and, thus, adding up to the highest number of patients of the just-referenced studies, we investigated the role of MD at diagnosis, assessed via the clinically widespread Breast Imaging-Reporting and Data System (BI-RADS), 5th Edition [17], as a potential treatment predictive biomarker for pCR in a larger dataset and across different St. Gallen surrogate subtypes of BC.

## Methods

### Cohort

**Fig. 1:**
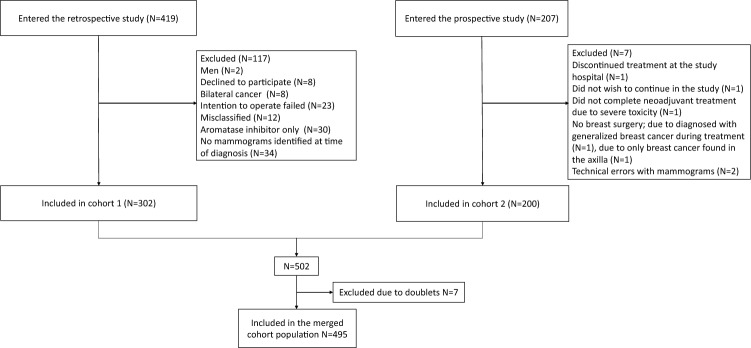
Patient flow chart

A total of 495 BC patients receiving NACT for BC were included in this study (Fig. 1). We combined two neoadjuvant cohorts: cohort 1, a retrospective cohort (“NeoMon”) of 302 patients receiving NACT for BC during 2005-2016 [15], and cohort 2, a prospective cohort (“NeoDense”) of 200 patients receiving NACT for BC during 2014–2019 [16] both at Skane University Hospital, Sweden. Seven patients were part of both cohorts and, hence, were excluded from cohort 1 before statistical analyses were performed. The details of the cohorts are previously described [15, 16]. Here, the two cohorts are briefly recapitulated and described, respectively. Only female patients treated with NACT and undergoing the intended breast surgery were included in the study. Patients’ characteristics were retrieved from medical charts for the retrospective cohort and from a study-specific patient questionnaire (filled in at the time of diagnosis) for the prospective cohort. Menopausal status at baseline was differently defined in the two cohorts, cohort 1: menopausal status was collected from patient records (perimenopausal patients were included in the postmenopausal group and when menopausal status was unknown, the patient was considered postmenopausal if > 55 years), cohort 2: defined according to self-reported menstrual history (patients > 1 year since the last period (not caused by birth control or recent pregnancy/breastfeeding) were considered postmenopausal); for the analyses of this study, patients ≥ 55 years were considered postmenopausal and otherwise premenopausal. Patients received their oncological treatment according to the same guidelines.

For both cohorts, the standard NACT contained three series of fluorouracil, epirubicin, and cyclophosphamide (FEC) or epirubicin and cyclophosphamide (EC) followed by three series of taxanes (docetaxel or paclitaxel) and, in the case of HER2 positive tumor, combined with HER2-blockade (trastuzumab/pertuzumab). In total, 87% of the patients received a chemotherapy regimen consisting of a combination of EC/FEC and docetaxel/paclitaxel. Additionally, a total of 10% of the patients received taxane-only NACT-regimen and a total of 2% of the patients received FEC/EC only. For the patients with HER2 positive tumors, the standard treatment differed between the two cohorts given the different time periods; in the retrospective cohort, a total of 97% (*n* = 90) received single (trastuzumab) anti-HER2 treatment, while the remainder (*n* = 3) received no anti-HER2 treatment. In the prospective cohort, a total of 94% (*n* = 45) of the patients with HER2 positive tumors received double (trastuzumab and pertuzumab) blockade, while the remainder (*n* = 3) received single (trastuzumab) HER2-blockade. Thus, overall, only 2% of patients with a HER2 positive tumor did not receive any anti-HER2 treatment at all.

Information about tumor pathology from the core biopsy at diagnosis and the surgical specimen following NACT was derived from clinical pathological reports. Following local and national guidelines defined identically for the two cohorts, tumor hormone receptor positivity was defined according to staining positive in > 10% of the tumor cells with IHC and HER2 status was defined as either 3+ with IHC and/or amplified with fluorescence in situ hybridization. The proliferation marker Ki67 was reported as a number from 0–100%, depending on the percentage of the tumor cells that stained positive. Ki67 > 20% was considered highly proliferative and Ki67 ≤ 20% was considered low proliferative. The same definition for pCR was used in both cohorts, i.e., the absence of any residual invasive cancer in the resected breast after surgery as well as all sampled regional lymph nodes following completion of NACT [18].

Patients were assigned to one of the following St. Gallen surrogate subtypes: luminal A-like (ER+, PR+/-, HER2-, Ki67 ≤ 20% “low”); luminal B-like (ER+, PR+/–, HER2-, Ki67 > 20% ”high”); HER2 positive (ER+/−, PR+/−, HER2+, any Ki67), or triple negative (ER-, PR-, HER2-, any Ki67).

### Mammograms

For cohort 1, we had access to digital vendor-processed mammograms at the time of diagnosis, while both digital unprocessed raw images and processed images were continuously assembled for cohort 2.

For cohort 1, the mammographic examination was performed at three different sites within Skane, Sweden on the following machines: Fujifilm, GE Healthcare, Philips Healthcare, and Siemens Healthineers. Using images in all available views and both breasts, categorization according to BI-RADS breast composition 5th Edition [17] was retrospectively done by an experienced specialist in radiology (HS) blinded to all patient and tumor characteristics. If the breasts were not of equal MD, the denser breast was used to categorize MD as is recommended by the BI-RADS guidelines [17]. In case of inflammatory BC (*n* = 10) the contralateral breast was used to categorize MD.

For cohort 2, mammograms were acquired on the following machines: GE, Philips, and Siemens. Categorization according to BI-RADS breast composition 5th Edition was done based on images in all available views from the contralateral breast at each radiological examination by the assigned breast radiologist in the clinic. The ipsilateral cancer-affected breast and the contralateral healthy breast showed good concordance in MD [16] in line with another study [19].

Information about tumor size (largest measurable diameter) was retrieved from clinical radiology reports (cohort 1) and study-specific forms (cohort 2). When both mammographic and ultrasound size estimation were available, the mean was used in the statistical analyses.

### Statistical analyses

We summarized baseline patient characteristics for the combined cohorts by both BI-RADS level and pCR. Categorical variables were summarized by counts and percentages, and continuous variables by median and interquartile range (IQR). We present estimates and IQR and have purposely left out* p*-values to avoid misinterpretation and over-belief in such significance testing [20]. Next, we estimated the association between BI-RADS and pCR as expressed by odds ratios (ORs) using logistic regression. Crude, uni-, and multivariable-adjusted models were used, including only the complete cases (*n* = 491). Adjustments were made for age, and age, menopausal, and body mass index (BMI), respectively. We modeled the association both overall and in subgroups defined by menopausal and St. Gallen surrogate subtype status. We categorized BI-RADS in two ways: as a vs. b, c, and d, and as a/b vs c/d. In the regression models, we used generalized estimating equations to account for within-hospital correlations. All analyses were carried out using SAS (SAS Institute Inc., Version 9.4 Car, NC, USA).

## Results

The patient and tumor characteristics of the 495 patients included in this study are presented according to BI-RADS category in Table 1. Most of the patients had intermediate dense breasts. A total of 84% of the patients were categorized as either BI-RADS b or c; only 5% and 11% were categorized as BI-RADS a and d, respectively. With increasing density according to BI-RADS, the median age and BMI declined expectedly and the proportion of premenopausal patients increased, whereas there was no association between tumor size and MD. Tumors in dense breasts (BI-RADS c and d) were, to a higher degree, ER and PR positive compared to tumors in less dense breasts. HER2 status was fairly equally distributed according to BI-RADS category. A total of 77% of the tumors were highly proliferative (Ki67 > 20%) and the proportions were similar in the different BI-RADS categories. The median tumor size was 30 mm (IQR 22–40) (cohort 1: 32.5mm (IQR 23.3–45.0) and cohort 2: 29 mm (IQR 20.0–37.5)). Positive axillary lymph node status was verified pre-NACT for 67% of the patients.

**Table 1:** Patients and tumor characteristics according to mammographic density at baseline

	Total cohort	BI-RADS a	BI-RADS b	BI-RADS c	BI-RADS d
Overall	495	25	192	226	52
Age, median (IQR)	53 (45 to 63)	59 (56 to 68)	58 (48 to 66)	50 (42 to 60)	46 (40 to 57)
BMI, median (IQR)	25 (23 to 29)	31 (28 to 36)	26 (24 to 29)	24 (22 to 27)	23 (21 to 26)
Tumor size (mm), median (IQR)	30 (22 to 40)	33 (24 to 39)	29 (20 to 38)	33 (24 to 43)	33 (21 to 40)
Menopausal status					
Premenopausal	269 (54.3%)	6 (24.0%)	78 (40.6%)	149 (65.9%)	36 (69.2%)
Postmenopausal	226 (45.7%)	19 (76.0%)	114 (59.4%)	77 (34.1%)	16 (30.8%)
Any births					
No children	69 (13.9%)	2 (8.0%)	18 (9.4%)	40 (17.7%)	9 (17.3%)
1 or more children	425 (85.9%)	23 (92.0%)	173 (90.1%)	186 (82.3%)	43 (82.7%)
Missing	1 (0.2%)	0 (0.0%)	1 (0.5%)	0 (0.0%)	0 (0.0%)
Ever hormone replacement therapy					
Yes	64 (12.9%)	4 (16.0%)	31 (16.1%)	25 (11.1%)	4 (7.7%)
No	427 (86.3%)	21 (84.0%)	157 (81.8%)	201 (88.9%)	48 (92.3%)
Missing	4 (0.8%)	0 (0.0%)	4 (2.1%)	0 (0.0%)	0 (0.0%)
ER					
Positive (> 10%)	299 (60.4%)	10 (40.0%)	113 (58.9%)	138 (61.1%)	38 (73.1%)
Negative (≤ 10%)	187 (37.8%)	15 (60.0%)	75 (39.1%)	83 (36.7%)	14 (26.9%)
Missing	9 (1.8%)	0 (0.0%)	4 (2.1%)	5 (2.2%)	0 (0.0%)
PR					
Positive (> 10%)	238 (48.1%)	10 (40.0%)	81 (42.2%)	117 (51.8%)	30 (57.7%)
Negative (≤ 10%)	247 (49.9%)	15 (60.0%)	107 (55.7%)	103 (45.6%)	22 (42.3%)
Missing	10 (2.0%)	0 (0.0%)	4 (2.1%)	6 (2.7%)	0 (0.0%)
HER2					
Positive	141 (28.5%)	7 (28.0%)	64 (33.3%)	56 (24.8%)	14 (26.9%)
Negative	338 (68.3%)	16 (64.0%)	121 (63.0%)	163 (72.1%)	38 (73.1%)
Missing	16 (3.2%)	2 (8.0%)	7 (3.6%)	7 (3.1%)	0 (0.0%)
Ki67					
High (> 20%)	381 (77.0%)	21 (84.0%)	152 (79.2%)	167 (73.9%)	41 (78.8%)
Low (≤ 20%)	54 (10.9%)	2 (8.0%)	17 (8.9%)	32 (14.2%)	3 (5.8%)
Missing	60 (12.1%)	2 (8.0%)	23 (12.0%)	27 (11.9%)	8 (15.4%)
St. Gallen					
Luminal A like	47 (9.5%)	1 (4.0%)	16 (8.3%)	28 (12.4%)	2 (3.8%)
Luminal B like	148 (29.9%)	5 (20.0%)	56 (29.2%)	66 (29.2%)	21 (40.4%)
HER2 positive	141 (28.5%)	7 (28.0%)	64 (33.3%)	56 (24.8%)	14 (26.9%)
Triple negative	121 (24.4%)	9 (36.0%)	42 (21.9%)	62 (27.4%)	8 (15.4%)
Missing	38 (7.7%)	3 (12.0%)	14 (7.3%)	14 (6.2%)	7 (13.5%)
Lymph node status					
Negative	79 (16.0%)	3 (12.0%)	30 (15.6%)	39 (17.3%)	7 (13.5%)
Positive	334 (67.5%)	17 (68.0%)	131 (68.2%)	148 (65.5%)	38 (73.1%)
Missing	82 (16.6%)	5 (20.0%)	31 (16.1%)	39 (17.3%)	7 (13.5%)

A total of 102 patients (21%) accomplished pCR (Table 2) (19% in the retrospective cohort and 23% in the prospective cohort). A total of 32%, 24%, 17%, and 15% of the BI-RADS a, b, c, and d patients, respectively, reached pCR. Age, menopausal status, BMI, tumor size, number of births, and use of hormone replacement treatment was fairly equally distributed between patients who accomplished pCR and those who did not. Patients accomplishing pCR more often had ER and/or PR negative tumors, HER2 positive tumors, and highly proliferative tumors. The highest proportion of pCR was seen in the HER2 positive subtype (54%), followed by the triple negative subtype (34%). None of the 47 patients with luminal A-like tumors accomplished pCR.

**Table 2:** Patient and tumor characteristics at baseline according to pathological complete response

	pCR	non pCR
Overall	102	393
BI-RADS		
BI-RADS a	8 (7.8%)	17 (4.3%)
BI-RADS b	47 (46.1%)	145 (36.9%)
BI-RADS c	39 (38.2%)	187 (47.6%)
BI-RADS d	8 (7.8%)	44 (11.2%)
Age, median (IQR)	54 (46 to 63)	53 (45 to 63)
BMI, median (IQR)	25 (23 to 29)	25 (23 to 29)
Tumor size (mm), median (IQR)	26 (20 to 35)	33 (23 to 40)
Menopausal status		
Premenopausal	53 (52.0%)	216 (55.0%)
Postmenopausal	49 (48.0%)	177 (45.0%)
Any births		
No children	10 (9.8%)	59 (15.0%)
1 or more children	92 (90.2%)	333 (84.7%)
Missing	0 (0 %)	1 (0.3%)
Ever hormone replacement therapy		
Yes	16 (15.7%)	48 (12.2%)
No	86 (84.3%)	341 (86.8%)
Missing	0 (0 %)	4 (1.0%)
ER		
Positive (> 10%)	28 (27.5%)	271 (69.0%)
Negative (≤ 10%)	73 (71.6%)	114 (29.0%)
Missing	1 (1.0%)	8 (2.0%)
PR		
Positive (> 10%)	17 (16.7%)	221 (56.2%)
Negative (≤ 10%)	84 (82.4%)	163 (41.5%)
Missing	1 (1.0%)	9 (2.3%)
HER2		
Positive	55 (53.9%)	86 (21.9%)
Negative	45 (44.1%)	293 (74.6%)
Missing	2 (2.0%)	14 (3.6%)
Ki67		
High (> 20%)	89 (87.3%)	292 (74.3%)
Low (≤ 20%)	3 (2.9%)	51 (13.0%)
Missing	10 (9.8%)	50 (12.7%)
St. Gallen		
Luminal A like	0 (0 %)	47 (12.0%)
Luminal B like	9 (8.8%)	139 (35.4%)
HER2 positive	55 (53.9%)	86 (21.9%)
Triple negative	35 (34.3%)	86 (21.9%)
Missing	3 (2.9%)	35 (8.9%)
Lymph node status		
Negative	23 (22.5%)	56 (14.2%)
Positive	66 (64.7%)	268 (68.2%)
Missing	13 (12.7%)	69 (17.6%)

**Fig. 2:**
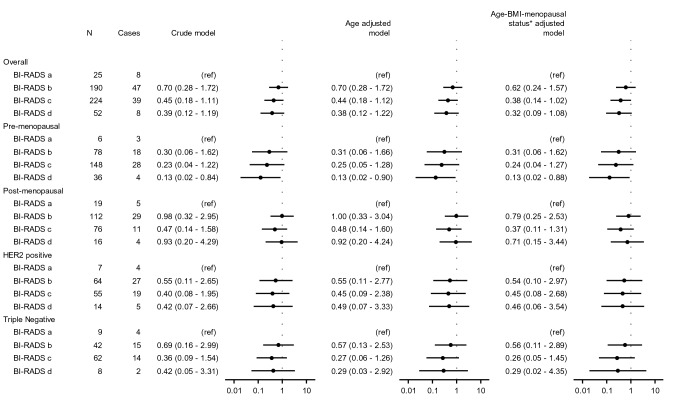
Forest plot: associations between mammographic density at baseline and pathological complete response following neoadjuvant chemotherapy (BI-RADS **a** vs. **b**, **c** and **d**, respectively). The following footnote should be written below the image: *Applicable to: “Overall”, “HER2 positive”, and “Triple negative”

A total of 491 patients had complete study data and were included in the logistic regression models (Fig. 2) addressing the association between MD assessed with BI-RADS and pCR following NACT in three different models. Overall, there was an inclination for the following: the higher the breast density, the lower the OR of accomplishing pCR; in comparison to patients with BI-RADS a, the ORs of accomplishing pCR in the multivariable-adjusted model were: BI-RADS b 0.62 (95% confidence interval (CI) 0.24–1.57), BI-RADS c 0.38 (95% CI 0.14–1.02), and BI-RADS d 0.32 (95% CI 0.09–1.08). For premenopausal patients, the corresponding ORs of accomplishing pCR in comparison to patients with BI-RADS a were: BI-RADS b 0.31 (95% CI 0.06–1.62), BI-RADS c 0.24 (95% CI 0.04–1.27), and BI-RADS d 0.13 (95% CI 0.02-0.88). Thus, in this model, the association was more pronounced when the premenopausal patients were analyzed separately, showing lower point estimates, although wider CI. When the cohort was dichotomized according to BI-RADS classification (a/b vs. c/d), patients with high MD had lower OR of accomplishing pCR (0.58 (95% CI 0.36–0.92) in comparison to patients with low MD (Supplementary Material 1).

During analysis of subgroups according to St. Gallen surrogate subtypes, due to no and only a few events of pCR in the luminal A-like and luminal B-like groups, no logistic regression was performed for these subgroups. No significant association between MD and the likelihood of accomplishing pCR following NACT was found for the HER2 positive and triple negative subtypes, separately (Fig. 2). However, these subgroup analyses did not contradict the main results; the point estimates for both HER2 positive and triple negative subtypes indicated a lower likelihood of accomplishing pCR with high MD (BI-RADS c and d) in comparison to BI-RADS a.

## Discussion

In this study of 495 BC patients from both a retrospective cohort and a prospective cohort, we found the following tendency: the higher the MD, the lower the likelihood of accomplishing pCR following NACT, most notably for premenopausal patients. The clinical interpretation is that MD, as assessed with BI-RADS, may be used as a predictive biomarker for NACT. Subgroup analysis based on St. Gallen surrogate subtypes resulted in imprecise estimates, though in line with the main results.

In our study, tumors in denser breasts (BI-RADS c and d) were more often ER and PR positive compared to tumors in less dense breasts; thus, luminal-like BC was overrepresented in dense breasts. The majority of all tumors were highly proliferative regardless of MD, probably related to the indication of NACT [21]. Scientific inconsistency exists regarding the association between tumor receptor status and MD [22]; however, larger tumor size and positive lymph node status are suggested to be linked to high MD [23]. MD changes throughout a woman’s life and is strongly associated with hormonal events; however, according to a review by Martin et al. most studies found no association between serum estrogen levels and MD in either pre- or postmenopausal women [24]. MD decreases with increasing age, with a steep decline during the perimenopausal period [24]—hence, our stratification according to menopausal status.

There might be several explanations as to why women with low MD could be more likely to accomplish pCR than their counterparts with denser breasts. Although much has been written about the biological links between MD and risk of BC, to our knowledge, no prevailing biological explanation has been presented regarding MD and response to NACT. Response to treatment is influenced not only by tumor characteristics but also by host factors, e.g., age and BMI; we believe that MD can be considered to be one of these host factors, representing the microenvironment of the surrounding breast tissue [14]. On a tissue level, the relationship of mitogens (e.g., insulin growth factor and prolactin) and mutagens (e.g., urinary malondialdehyde, an indicator of oxidative stress) makes MD a proliferative and pro-inflammatory environment [24, 25]. We believe that the same mechanism responsible for tumor initiation and tumor growth may be responsible for a poorer response to treatment in dense breasts. Also, the higher pressure gradient and the higher number of molecules to interact with in a dense breast in comparison to a less dense breast can hypothetically obstruct the delivery of the systemically administrated drug to the tumor site [26–28]. In a study of triple negative BC receiving adjuvant chemotherapy, high MD was associated with higher rates of locoregional recurrence [29]. In a review, Shawky et al. found an adverse impact of high MD on local relapse; however, the emphasis was on radiotherapy, with only a minority of the patients receiving chemotherapy [30]. It is tempting to speculate on whether our results from a mixed BC subtype cohort are also applicable in the adjuvant setting, i.e., if women with dense breasts might experience an inferior locoregional effect of the adjuvant chemotherapy treatment, resulting in higher rates of locoregional recurrence.

Only a few patients had tumors classified as luminal A-like (*N* = 47), of which none accomplished pCR. A total of 148 patients were assigned to the luminal B-like group; due to the underlying indication for NACT, we consider these and similar numbers to be expected. At present, according to national guidelines, it is recommended that NACT be offered to patients with large tumors (T3/T4), patients with positive lymph node status in the axilla, and patients with tumors larger than 20 mm and, simultaneously, other risk factors, e.g., triple negative tumor or HER2 positivity [9, 10]. Due to no or only a few (*n* = 9) events of pCR in patients with luminal A-like or luminal B-like, respectively, it was not possible to conduct the logistic regression within the luminal-like subtypes. It is widely known that tumors of luminal-like subtypes have a lower rate of pCR following NACT in comparison to HER2 positive and triple negative subtypes [12]. Our results indicate a response rate of < 2 % and 6% for luminal A-like and luminal B-like, respectively—and, thus, relatively low rates of pCR.

Some study limitations must be discussed. The patients received their oncological treatment between 2005 and 2019, and the recommended treatment changed during this wide timespan. The most apparent differences were more stringency with regard to the recommended treatment of chemotherapy (three cycles of FEC/EC combined with three cycles of taxanes) and the fact that double HER2-blockade was given to the patients treated in the more recent years. One shortcoming of this study is that information about the indication for NACT is lacking; thus, one can only hypothesize regarding a potential shift in indication in the two different cohorts. One would assume that a larger proportion of patients received NACT in a down-staging purpose in the early years, although this is not reflected in the only marginally larger tumors seen in the retrospective cohort. A larger proportion of patients in the prospective cohort had axillary lymph node metastases, most likely due to a shift in indication for NACT. The rates of pCR (of patients included in the logistic regression models) in the two cohorts were rather similar and consistent with the previous literature [12]. We classified the patients as postmenopausal when age ≥ 55 years; hence, some patients might have been misclassified in terms of menopausal status. However, this does not influence the result in the unstratified, unadjusted logistic regression model. We did not have access to raw data mammograms for cohort 1, ruling out MD assessment by many automatic volumetric software programs. However, our choice of method, BI-RADS, is a widely clinically used method and our results are, thus, easier to applicate in a clinical setting. Given the somewhat inconsistent results of our previous studies [15, 16], our present study shows point estimates slightly closer to 1 and narrower CI, resulting in a more precise conclusion

In conclusion, it seemed as though patients with higher MD at baseline were less likely to reach pCR after NACT compared to patients with less dense breasts. This finding was more pronounced in premenopausal women. Larger studies are needed to perform subgroup analyses based on surrogate subtype to make MD useful as a predictive biomarker in the neoadjuvant clinical setting.

## Supplementary Information

 Below is the link to the electronic supplementary material.

Supplementary Material 1. Forest plot: Associations between mammographic density at baseline and pathological complete response following neoadjuvant chemotherapy (BI-RADS a/b vs. c/d) (PDF 105 kb)

## Data Availability

The datasets used and/or analyzed in the current study are available from the corresponding author on reasonable request.

## Abbreviations

BC: Breast cancer
MD: Mammographic density
ER: Estrogen receptor
PR: Progesterone receptor
HER2: Human epidermal growth factor receptor 2
IHC: Immunohistochemistry
NACT: Neoadjuvant chemotherapy
pCR: Pathological complete response
BI-RADS: Breast imaging reporting and data system
FEC: Fluorouracil, epirubicin and cyclophosphamide
EC: Epirubicin and cyclophosphamide
IQR: Interquartile range
OR: Odds ratio
BMI: Body mass index
CI: Confidence interval
