# Supplementary figures and images for: Mammographic density as an image-based biomarker of therapy response in neoadjuvant-treated breast cancer patients
Source: Cancer Causes Control. 2020 Dec 30;32(3):251–60. doi: 10.1007/s10552-020-01379-w (PMC7870759; doi:10.1007/s10552-020-01379-w)

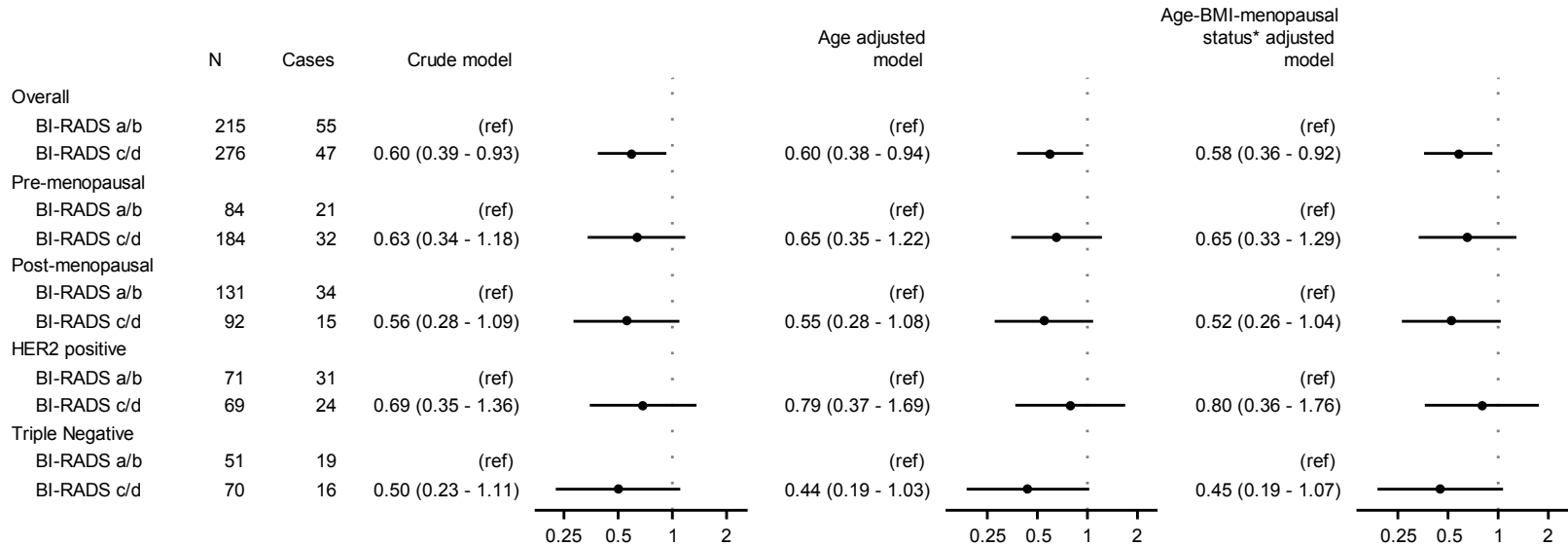

\*Applicable to: "Overall", "HER2 positive", and "Triple negative"

Supplement: Supplementary file 1 — Supplementary Material 1. Forest plot: Associations between mammographic density at baseline and pathological complete response following neoadjuvant chemotherapy (BI-RADS a/b vs. c/d) (PDF 105 kb) [file 10552_2020_1379_MOESM1_ESM.pdf]
